# Does Herbivorous Fish Protection Really Improve Coral Reef Resilience? A Case Study from New Caledonia (South Pacific)

**DOI:** 10.1371/journal.pone.0060564

**Published:** 2013-04-05

**Authors:** Laure Carassou, Marc Léopold, Nicolas Guillemot, Laurent Wantiez, Michel Kulbicki

**Affiliations:** 1 Research Unit 227 (Coreus), Institut de Recherche pour le Développement (IRD), Nouméa, New Caledonia; 2 Research Unit 227 (Coreus), Institut de Recherche pour le Développement (IRD), c/o Vanuatu Fisheries Department, Port-Vila, Vanuatu; 3 Research Unit LIVE (EA4243), University of New Caledonia, Nouméa, New Caledonia; 4 Aquarium des Lagons, Nouméa, New Caledonia; 5 Research Unit 227 (Coreus), Institut de Recherche pour le Développement (IRD), c/o Laboratoire Arago, Banyuls-sur-Mer, France; Swansea University, United Kingdom

## Abstract

Parts of coral reefs from New Caledonia (South Pacific) were registered at the UNESCO World Heritage list in 2008. Management strategies aiming at preserving the exceptional ecological value of these reefs in the context of climate change are currently being considered. This study evaluates the appropriateness of an exclusive fishing ban of herbivorous fish as a strategy to enhance coral reef resilience to hurricanes and bleaching in the UNESCO-registered areas of New Caledonia. A two-phase approach was developed: 1) coral, macroalgal, and herbivorous fish communities were examined in four biotopes from 14 reefs submitted to different fishing pressures in New Caledonia, and 2) results from these analyses were challenged in the context of a global synthesis of the relationship between herbivorous fish protection, coral recovery and relative macroalgal development after hurricanes and bleaching. Analyses of New Caledonia data indicated that 1) current fishing pressure only slightly affected herbivorous fish communities in the country, and 2) coral and macroalgal covers remained unrelated, and macroalgal cover was not related to the biomass, density or diversity of macroalgae feeders, whatever the biotope or level of fishing pressure considered. At a global scale, we found no relationship between reef protection status, coral recovery and relative macroalgal development after major climatic events. These results suggest that an exclusive protection of herbivorous fish in New Caledonia is unlikely to improve coral reef resilience to large-scale climatic disturbances, especially in the lightly fished UNESCO-registered areas. More efforts towards the survey and regulation of major chronic stress factors such as mining are rather recommended. In the most heavily fished areas of the country, carnivorous fish and large targeted herbivores may however be monitored as part of a precautionary approach.

## Introduction

Coral reefs are one of the ecosystems the most susceptible to climate change [1], [2]. Among other causes, this is due to 1) the high sensitivity of reef-building corals to rising water temperature, leading to bleaching events, and 2) their tropical distribution that makes them susceptible to physical destruction by hurricanes [3], [4]. These climate-induced disturbances, combined with other stress factors such as fishing [5], [6], predator outbreaks [7], [8], diseases [9], [10], or pollution [11], [12] have resulted in a significant decline of coral reefs worldwide during the last decades [3], [13]. These observations have led to pessimistic predictions for the future persistence of coral-dominated ecosystems [14]–[16].

In many instances, massive coral mortality observed after bleaching events or hurricanes has been followed by a shift in the dominance of benthic organisms [17]. These phase shifts have been observed in degraded systems virtually everywhere in the tropics, where many coral-dominated reefs were replaced by reefs dominated by macroalgae, soft corals, sponges, sea urchins, or ascidians [18]. Macroalgae-dominated reefs appear as the most frequent alternate state observed in degraded coral reefs worldwide [17]. For management purposes, this alternate state is not desired because, besides lower species diversities, reefs dominated by macroalgae provide less ecosystem goods and services than coral-dominated reefs [3]. Management strategies that may improve the resilience of coral reefs to climate change, in particular by mitigating the appearance and persistence of coral-macroalgae phase-shifts, and by facilitating the recovery of corals after natural disasters (e.g., bleaching events, hurricanes), are thus required.

New Caledonia, South Pacific, is one of the largest coral systems in the world, with 4,537 km^2^ of coral reef formations. The lagoon area extends over 31,336 km^2^ and is surrounded by a complex barrier reef of approximately 1,500 km in linear distance [19]. This exceptional coral reef complex provides a high diversity of habitats and species: 310 species of corals [20], 438 species of macroalgae [21] and 1,851 species of fish [22]. Reefs from New Caledonia have been mildly impacted by hurricanes and bleaching events as compared to reefs from the Caribbean or the Indian Ocean [13]. However, hurricane Erica damaged the west coast of the main island in 2003 [23], [24]. A single bleaching event was reported in 1996, locally affecting corals from the southwest lagoon around the capital city of Nouméa, down to a depth of 60 m [25].

About 2,300 km^2^ and 13,400 km^2^ of New Caledonia’s reefs and lagoons, respectively, were registered at the UNESCO World Natural Heritage list in 2008, as one of the 196 most pristine and unique natural sites in the world. Management strategies aiming at preserving the exceptional ecological value of these reefs in the context of climate change are thus currently being considered by public authorities. In particular, the fishing ban of all herbivorous fish species has been proposed as a strategy to enhance coral recovery in case of climate-induced disturbances in areas registered at the UNESCO Heritage list (hereafter “UNESCO areas”). This option relies on the assumption that higher density, biomass and/or diversity of herbivorous fish resulting from their exclusive protection would increase fish grazing on macroalgae, therefore contributing to the regulation of the occurrence and persistence of coral-macroalgae phase shifts [3], [26]. The appropriateness of such a management strategy remains to be critically evaluated, based on local observations but also in the light of current debate about the ability of local management decisions to mitigate the effects of global-scale sources of coral mortality and the persistence of alternate states of reef systems [27]–[30].

The objective of this study was to assess the relevance of an exclusive protection of herbivorous fish in order to promote coral reefs resilience to climate-induced disturbances (i.e., hurricanes and beaching events) in UNESCO areas of New Caledonia. A two-phase approach was developed to address this objective. In phase 1, corals, macroalgae and herbivorous fish communities were examined in a variety of biotopes from reefs submitted to different levels of fishing pressure across New Caledonia archipelago. The influence of current fishing pressure on herbivorous fish communities and coral and macroalgal covers in the country was analyzed. In phase 2, results from phase 1 were challenged in the context of a global synthesis of the influence of herbivorous fish protection on the response of reef benthic communities to hurricanes and bleaching events. The literature on coral recovery and macroalgal development relative to corals observed specifically after hurricanes and/or bleaching events in protected and unprotected areas throughout the world was reviewed, including observations of the effects of hurricane Erica on the New Caledonian reefs. Insights from both local and global analyses were then put into perspective, in order to formulate recommendations for the management of New Caledonia’s UNESCO areas and, more generally, well-preserved Indo-Pacific reefs supporting high coral and fish diversity.

## Results

### Influence of Reef Protection Status on Coral Recovery and Macroalgal Development: The Case Study of New Caledonia

**Figure 1:**
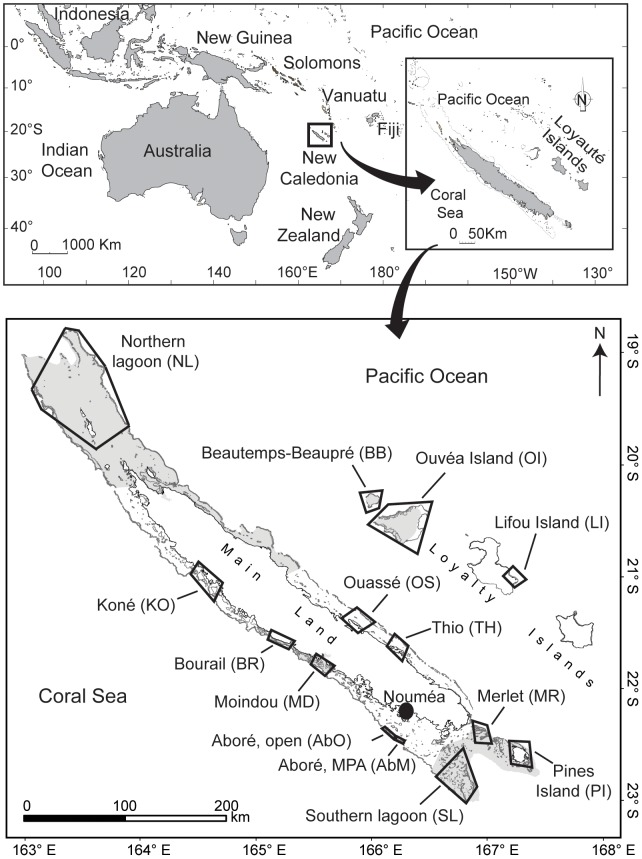
Location of the 14 study sites in New Caledonia, Southwest Pacific. Site codes are given in capital letters between brackets. Grey areas were registered at the UNESCO World Heritage list in 2008. “MPA” = marine protected area. “Open” = open fishing area.

A total of 111 species of herbivorous fish were observed in the 14 sites surveyed in New Caledonia (Fig. 1; Appendix S1a). These 111 species were classified into five groups, as a function of their size, diet and fishing status (Table 1; Appendix S1a). All 44 fished species were large and included 32 species feeding on microalgae only (“Large fished microalgae feeders”) and 12 species consuming both macro- and microalgae (“Large fished macroalgae feeders”) (Appendix S1a; Table 1). Few unfished species were large (“Large unfished herbivorous”, N = 12) but the majority were small (“Small unfished herbivorous”, N = 55). The diet of 65 out of these 67 unfished species was composed of microalgae only, except that of one large species, *Siganus puellus*, and one small species, *Siganus spinus*, which both also consumed macroalgae (Appendix S1a; Table 1). Therefore, 14 fish species (12.6% of herbivorous fish species) consumed macroalgae, including the two latter unfished Siganidae and 12 large fished species: five Acanthuridae (*Naso brachycentron*, *N. brevirostris*, *N. lituratus*, *N. tonganus*, *N. unicornis*), three Kyphosidae (*Kyphosus cinerascens*, *K. sydneyanus*, *K. vaigiensis*), and four Siganidae (*Siganus argenteus*, *S. fuscescens*, *S. lineatus*, *S. woodlandi*) (Appendix S1a).

**Table 1:** Classification of herbivorous fish species from New Caledonia into five groups based on species diet, fishing status, and size [31]–[36].

Group #	Label	Number of species
1	All herbivorous	111
2	Large unfished herbivorous	12
3	Small unfished herbivorous	55
4	Large fished macroalgae feeders	12
5	Large fished microalgae feeders	32

Groups 1 and 4 include species consuming both macro- and microalgae.

Group 5 includes species consuming microalgae only. Most species from groups 2 and 3 consume microalgae only, except two species (see results).

Refer to Appendix S1a for a complete species list.

Among the 14 sites surveyed, six were not subjected to fishing due to their protection status or their remoteness. The eight remaining sites were characterized by low (N = 4) to moderate (N = 4) fishing pressure (Fig. 1; Table 2). Fishing pressure did not affect the absolute biomass, density and diversity of fished herbivores, but positively impacted the biomass, density and diversity of fish groups relative to the whole fish community (Table 3; Appendix S1c). However, when related to herbivorous species only, the relative biomass, density and diversity of most fished groups were not significantly affected by fishing (Table 3; Appendix S1c). Fishing also decreased the size of herbivorous fish, particularly that of large fished microalgae feeders and small unfished herbivores (Table 3; Appendix S1c). No difference between biotopes was detected among the variables tested regardless of fishing pressure, except for macroalgal cover which was higher in fringing reefs (Table 3; Appendix S1c). The interaction between biotope and fishing pressure was not significant on any of the variables examined.

**Table 2:** Characteristics of the 14 sites sampled in New Caledonia between 2004 and 2008, in terms of protection status, UNESCO labeling, human population, fishing pressure, and underwater ecological surveys.

	NL	BB	OI	LI	OS	TH	PI	MR	SL	AbM	AbO	MD	BR	KO
**Protection status at the time of sampling**	Open	Open	Open	Open	Open	Open	Open	MPA	Open	MPA	Open	Open	MPA	Open
**UNESCO registration in 2008**	Yes	Yes	Yes	No	No	No	Yes	Yes	Yes	No	No	No	No	No
**Coastal human population × 1,000 inhab.**	0.0	0.0	4.0	2.0	3.0	1.0	2.0	0.2	0.0	100.0	100.0	5.0	5.0	9.0
**Fishing area (km^2^)**	0.0	0.0	710.6	35.3	483.0	555.0	741.4	0.0	0.0	0.0	1889.0	281.5	0.0	621.4
**Human pressure (inhab.km^−2^)**	0.0	0.0	5.6	56.6	6.2	1.8	2.7	0.0	0.0	0.0	52.9	17.8	0.0	14.5
**Fishing pressure category**	None	None	Low	Mod.	Low	Low	Low	None	None	None	Mod.	Mod.	None	Mod.
**Number of stations in**														
**Fringing reefs**	4	na	2	3	6	5	na	na	na	na	na	3	na	na
**Lagoon patch reefs**	12	1	1	7	4	4	6	na	5	na	na	3	5	8
**Inner barrier reefs**	6	5	8	na	6	4	7	14	3	24	12	6	4	19
**Outer barrier reefs**	7	4	10	2	8	14	10	7	7	na	na	9	6	7

See Materials and Methods for details on the determination of fishing pressure categories. “Mod.” = moderate, “inhab.” = inhabitants, “MPA” = Marine Protected Area, “na” = not applicable. Sites codes are depicted in Fig. 1.

**Table 3:** Results of factorial ANOVA testing for the effect of biotope (d.f = 3), fishing pressure (hereafter “fishing”, d.f. = 2) and their interaction (d.f. = 6) on coral reef benthic and fish communities in New Caledonia.

Variables	Factor	*P*
**Substrate cover (%)**		
Macroalgae cover	Biotope	*
	Fishing	**
**Biomass (g.m^−2^)**		
Small unfished herbivorous	Fishing	***
**Biomass (% total fish)**		
All herbivorous	Fishing	****
Large unfished herbivorous	Fishing	*
Small unfished herbivorous	Fishing	****
Large fished macroalgae feeders	Fishing	****
Large fished microalgae feeders	Fishing	****
**Biomass (% herbivorous fish)**		
Large fished microalgae feeders	Fishing	*
**Density (% total fish)**		
All herbivorous	Fishing	****
Small unfished herbivorous	Fishing	****
Large fished macroalgae feeders	Fishing	**
Large fished microalgae feeders	Fishing	****
**Diversity (number of species)**		
Small unfished herbivorous	Fishing	*
**Diversity (% total fish)**		
All herbivorous	Fishing	****
Large unfished herbivorous	Fishing	****
Small unfished herbivorous	Fishing	****
Large fished macroalgae feeders	Fishing	****
Large fished microalgae feeders	Fishing	****
**Diversity (% herbivorous fish)**		
Small unfished herbivorous	Fishing	*
Large fished microalgae feeders	Fishing	*
**Size (last quartile, cm)**		
All herbivorous	Fishing	****
Small unfished herbivorous	Fishing	*
Large fished microalgae feeders	Fishing	***

Only significant effects are reported for clarity, but see Appendix S1c for a complete report, including all relationships tested with corresponding post-hoc tests for the effect of fishing pressure. Groups of herbivorous fish are detailed in Appendix S1a. “****”: *P*<0.001, “***”: *P*<0.005, “**”: *P*<0.01, “*”: *P*<0.05.

Coral cover was similar for all levels of fishing pressure, whereas macroalgae were significantly less abundant in unfished areas (Table 3; Appendix S1c). However, macroalgal cover was not correlated with the biomass, density and diversity of macroalgae feeders whatever the biotope or level of fishing pressure considered (*P*>0.05 in all cases). No significant correlation between coral cover and macroalgal cover was found in any of the biotope or level of fishing pressure (*P*>0.05 in all cases).

### Influence of Reef Protection Status on Coral Recovery and Macroalgal Development: A Global Analysis

**Figure 2:**
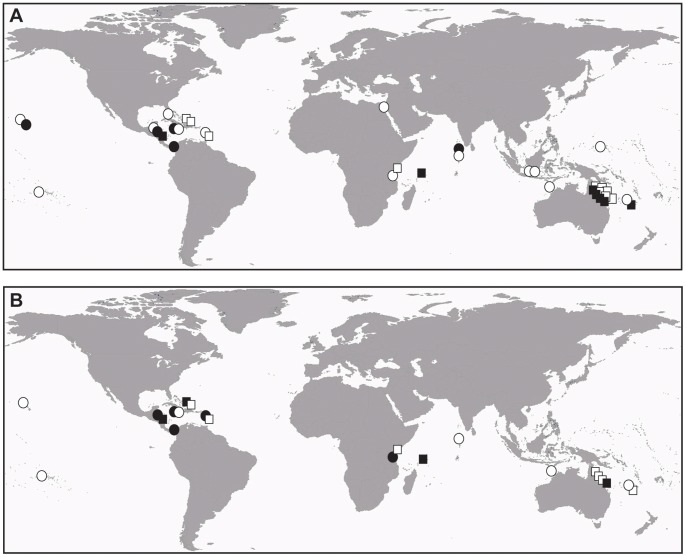
Distribution of (A) coral recovery (*CR*) and (B) macroalgal relative development (*MD*) after climatic disasters. Circles represent open fishing areas. Squares represent marine protected areas. White labels represent positive *CR* values (*CR*>0) and the absence of relative macroalgal development after the event (*MD* = “−”). Black labels represent negative *CR* values (*CR*<0) and a development of macroalgae relative to corals after the event (*MD = *“+”).

A total of 27 references from the literature were reviewed for analyzing the effect of reef protection status on coral recovery and relative macroalgal development after climate-induced disturbances on a global perspective (Table 4; Appendix S2). The 27 selected references included 36 case studies, which encompassed the four main regions of coral reef geographical range: the Red Sea (1 study), the Indian Ocean (5 studies), the Atlantic Ocean (11 studies) and the Pacific Ocean (19 studies, including two from New Caledonia) (Fig. 2; Table 4). The survey durations ranged from one to 22 years: less than five years in 17 studies, between five and 10 years in 13 studies, and more than 10 years in six studies (refer to Appendix S2 for raw data).

**Table 4:** Indices of coral recovery (*CR*) and macroalgae development relative to corals (*MD*) after climatic disturbances based on a literature review.

Location	Protection status		Disturbance	*CR*	*MD*	Reference
	MPA	Open				
**ATLANTIC OCEAN**						
**Belize**	X		H+B	−0.06	“+”	[31]
		X	H+B	0.02	“+”	[31]
		X	H+B	−0.82	na	[32]
**Florida Keys**		X	H+B	0.22	na	[33]
**Jamaica**						
Discovery Bay		X	H+B	−0.09	“+”	[34]
Daily Bull Reef		X	H+B	0.26	“−”	[35]
**Panama**						
San Blas Islands		X	B	−0.15	“+”	[36]
**St Lucia**	X		H	0.10	“−”	[37]
		X	H	0.12	“+”	[37]
**Virgin Islands**						
St Croix, Buck Island	X		H	0.43	“−”	[38]
St John, Yawsi Point	X		H	0.22	“+”	[39]
**RED SEA**						
**Arabic Gulf, Dubai**		X	B	0.15	na	[40], [41]
**INDIAN OCEAN**						
**Kenya**	X		B	0.55	“−”	[42]
		X	B	0.29	“+”	[42]
**Maldives**		X	B	−0.20	na	[43]
		X	B	0.62	“−”	[44]
**Seychelles, Cousin Island**	X		B	−0.16	“+”	[45]
**PACIFIC OCEAN**						
**Australia, Great Barrier Reef**						
Middle Island	X		B	1.78	“−”	[46]
Halfway Island	X		B	1.83	“−”	[46]
Barren Island	X		B	1.96	“−”	[46]
North Keppel Island	X		B	0.20	“+”	[46]
Capricorn Bunker, Swain	X		H	1.10	na	[47]
Heron Island	X		B	−1.60	na	[48]
Heron Island, inner flat	X		H	−0.68	na	[49]
Heron Island, exposed pools	X		H	0.42	na	[49]
Heron Island, protected crest	X		H	−0.10	na	[49]
Heron Island, exposed crest	X		H	−0.07	na	[49]
**Australia NW, Scott Reef**		X	B	0.27	“−”	[50]
**Hawai’i**						
Oahu		X	H	0.04	“−”	[51]
West coast		X	H	−0.01	na	[52]
**Indonesia, 1000 Islands**						
South Pari		X	B	0.67	na	[53]
South Tikus		X	B	1.08	na	[53]
**Micronesia, Palau**		X	B	0.36	na	[54]
**New Caledonia**						
Southwest lagoon	X		H	−0.59	“−”	[23]
Northwest lagoon		X	H	0.80	“−”	[24]
**Polynesia, Morea**		X	H+B	0.15	“−”	[55]

“H” = hurricane, “B” = bleaching, “MPA” = Marine Protected Area, “Open” = open fishing area, “na” = not available.

Raw data used for CR and MD calculations are provided in Appendix S2, and calculation formulae are provided in the Materials and Method section.

On a global scale, there was no apparent geographical trend in the distribution of coral recovery indices (*CR*; Fig. 2). The proportion of case studies for which corals did not appear to recover after the disturbances (*CR*<0) was indeed globally similar in all geographical regions, varying from 31.6% in the Pacific, 36.4% in the Caribbean, to 40% in the Indian Ocean (Fig. 2; Table 4). The proportion of case studies for which macroalgae developed relative to corals after the disturbances (*MD* = “+”) was more variable depending on the geographical region, ranging from 11.1% in the Pacific, 50% in the Indian Ocean, to 66.7% in the Caribbean (Fig. 2; Table 4).

**Figure 3:**
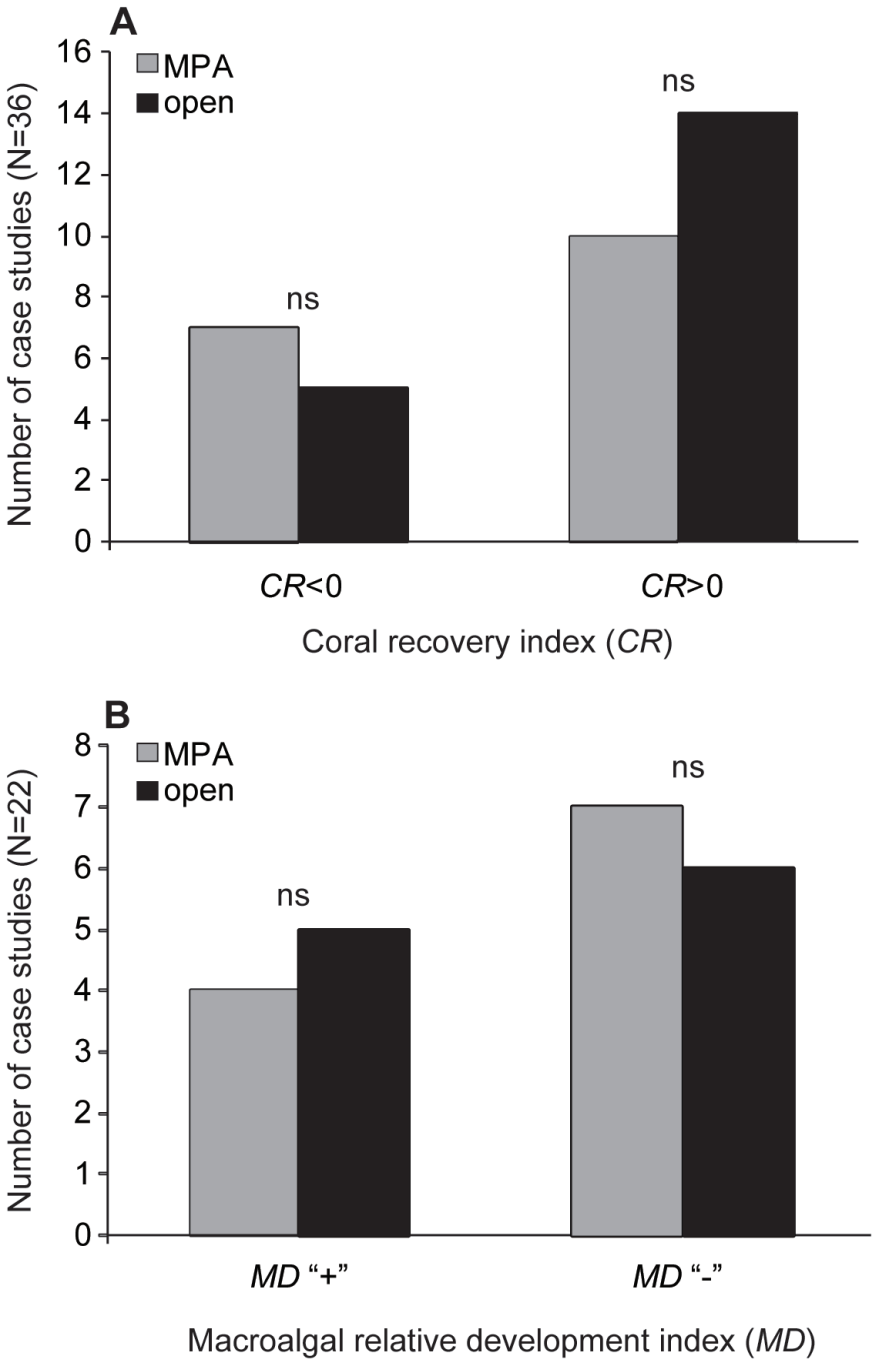
Influence of reef protection on (A) coral recovery (*CR*) and (B) macroalgal relative development (*MD*). Significance levels refer to Mann-Whitney tests of the effect of reef protection (two categories) on *CR* quantitative values and *MD* levels. “ns” = not significant, “MPA” = marine protected areas, “open” = open fishing areas.

Neither *CR* nor *MD* indices was related to the protection status of the reefs examined. A negative coral recovery (*CR*<0) was observed in 33.3% of the case studies, among which 58.3% had been conducted in marine protected areas (MPAs) and 41.7% in open reefs (Table 4). Macroalgae did develop relative to corals (*MD* = “+”) in 40.9% of the cases studies, among which 44.4% had been conducted in MPAs and 55.6% in open reefs (Fig. 2; Table 4). The two indices were not statistically different between MPAs and open reefs (Mann-Whitney tests, U = 159 and 55 for *CR* and *MD*, respectively; *P*>0.05 in both cases; Fig. 3). A similar pattern was observed at the scale of New Caledonia, where corals recovered better after hurricane Erica in open reefs from the northwest lagoon than in the MPA from the southwest lagoon. Moreover, macroalgae did not develop in either case (Table 4).

*CR* values also appeared unrelated to the pre-disturbance coral and macroalgal covers (*C_t0_* and *M_t0_*; *P*>0.05 in both cases), the initial decline in coral cover observed immediately after the disturbance (*ID*, *P*>0.05), or the period of time during which coral recovery was measured (*t_2_*–*t*_1_, *P*>0.05). The *CR* index was logically closely related to the post-disturbance estimate of coral growth (*PD*, r^2^ = 0.929; *P*<0.001). The latter represented the variation of coral cover several months to years after the climatic disturbance, a period during which many coral colonies grew back if no other major disturbance occurred.

## Discussion

### Status of New Caledonia’s Reefs

Out of the 111 species of herbivorous fish censused in New Caledonia, only 14 were shown to consume macroalgae. This is consistent with previous findings reporting that only a few species of fish are able to actively remove macroalgae on coral reefs, these species being highly variable across the world [56]–[58]. Among these 14 species, 12 are actually fished in New Caledonia. The size, biomass, density and diversity of these species showed little variation between levels of fishing pressure, providing evidence that fishing does not noticeably affect macroalgae feeders in New Caledonia at the present day. In addition, several of these species, i.e., *Siganus argenteus*, *S. fuscescens*, *S. woodlandi*, *Kyphosus* spp., are often found in non-reef habitats, in particular algal beds, and a large proportion of the juveniles of at least *S. argenteus* and *S. fuscescens* are frequently found in seagrass and algal beds [59]. As seagrass and algal beds are less targeted by fishers than coral reefs in New Caledonia [60], this could favor the persistence of large populations of these species.

Although herbivorous fish represent about 30% [60] to 50% [61] of estimated reef fish catches in New Caledonia, the biomass, density and diversity of each herbivorous group relative to the whole fish community were actually higher in the most heavily fished sites. This effect on the structure of the fish communities strongly suggests that fishing primarily affects fish of higher trophic levels in New Caledonia, and that an exclusive protection of herbivorous fish would unlikely significantly increase algal grazing in coral reefs. Furthermore, overall fishing pressure on reef fish resources is low in New Caledonia, where annual reef fish catch rate was estimated at about 200 kg.km^−2^ though higher levels have been observed close to densely-populated areas [62]. This would represent 0.1 to 0.6% of the fish biomass in the surveyed reef sites (Appendix S1b) and is not expected to increase in the areas located far from urban centers in the short term. Additionally, herbivores represent a large proportion of the fish biomass on reefs in New Caledonia, this proportion varying between 40 and 60% depending on sites (see also [63]–[66]) as in most reef fish assemblages in the Indo-Pacific [67]. As many species of the exploited herbivorous fish are fast growers (e.g., Scaridae, Siganidae), have a rapid initial growth (e.g., Acanthuridae) [68], or live in reef and non-reef habitats [59], [63], [69], it is likely that these fish would be rather resilient compared to most carnivorous or piscivorous species.

Strong negative correlations between coral and macroalgal covers were generally observed in relatively degraded reef systems, where a combination of high fishing pressure, intense coastal development, and/or frequent disturbances (i.e., hurricanes, bleaching) have resulted in a significant depletion of coral cover and the appearance of macroalgae-dominated alternate states [26], [70]–[74]. However, New Caledonia is considered a generally healthy reef system, as highlighted, for example, by the very low macroalgal cover across the 14 surveyed sites. The higher macroalgal cover observed in fringing reefs compared to other biotopes may be attributable to increasing oligotrophic waters from the coast to the barrier [75], [76]. The overall low fishing pressure and healthy status of coral reefs in New Caledonia, particularly in UNESCO areas, may then partly explain that no correlation was found between coral and macroalgal covers in the present study.

### A Larger Perspective

Published observations of the effect of the last serious climatic disturbance observed in New Caledonia in 2003 further emphasized the absence of any influence of protection on coral reef resilience to climatic disturbances in this region [23], [24]. These results were consistent with our global-scale analysis which showed that coral recovery or relative macroalgal development observed specifically after climate-induced disturbances were not related to reef protection status at a global scale. Darling et al. (2010) [77] also reported that the decline of live coral cover after the 1998 bleaching event in Kenya was not accelerated in fished areas compared to protected areas. Similarly, McClanahan (2008) [78] observed a slower coral recovery within fisheries closures than in unmanaged reefs in 12 sites from Kenya. Other studies conducted at a variety of spatial and temporal scales highlighted that reef protection, through the implementation of MPAs in particular, did not prevent coral loss in several locations in the south Pacific [79], the Indian Ocean [28], or the Atlantic [80]. Mora et al. (2008) [81] also concluded that, on a world-wide scale, although MPAs effectively increased the biomass of fish populations, they did not modify the patterns of change observed for coral reef builders and macroalgae. Conversely, several studies showed that MPAs can help in preventing coral loss and enhancing coral recovery (e.g., [82], [83]), and numerous experimental studies have advocated the protection of herbivorous fish for promoting coral reefs resilience to climate change (e.g., [26], [84]–[86]). These contradictory conclusions are representative of current debates on the ability of local management decisions to mitigate the effects of global-scale sources of coral mortality and the persistence of alternate states of reef systems [27]–[30], [87].

Discrepancies observed between studies may come from either the difficulty to translate results from small-scale experiments to processes occurring at larger scales in nature [82], [87] or context-dependent factors (e.g., geographical area, reef geomorphology, level of reef degradation, fishing pressure). Conversely to our study, many experimental studies that have shown a positive effect of MPAs on coral reef resilience to climatic disturbances focused on a few species of herbivorous fish only, generally large Scaridae which are heavily fished in the Caribbean and the Indian Ocean (e.g., [26], [82], [84]). Observed patterns of change in coral cover within MPAs may also vary depending on the species of corals and the spatial scale examined [82]. Similarly, Selig and Bruno (2010) [83] documented a significant effect of MPAs in mitigating coral loss at a global scale, but also emphasized a difference in MPAs benefits between geographical areas. In the Indo-Pacific in particular, the authors acknowledged that the beneficial effect of MPAs in decreasing rates of coral loss has been interrupted after the large-scale 1998 bleaching event. This observation confirmed that, in some areas, MPAs may not be able to protect corals from broad-scale natural disturbances such as ocean warming, large storms or disease outbreaks, a conclusion consistent with our and other studies [27], [28], [88], [89]. Furthermore, the global-scale analysis from Selig and Bruno (2010) [83] included a very large number of sites characterized by highly variable contexts (i.e., different fishing pressure, reef degradation status, other chronic stress factors). Detailed site-specific observations, such as those provided in the present study in New Caledonia, appear more appropriate to set local fisheries management rules.

Additionally, the hypothesis linking decreasing coral reef resilience to overfishing of herbivorous fish was mainly based on research carried out in the Caribbean, but its applicability to other biogeographic regions remains uncertain. Roff and Mumby (2012) [90] found that coral reef resilience in the context of climate change was expected to be higher in the Indo-Pacific than in the Caribbean or the Indian Ocean, due to, among other causes, 1) a higher diversity of coral species, resulting in more diversified responses to heat stress and physical destruction, 2) higher herbivorous fish biomass and diversity, 3) higher abundance of other herbivores such as sea urchins, and 4) lower macroalgal growth and recruitment rates. The composition of the regional herbivore species pool could also play a role as the number of available species may be very different from one region to another. For instance, in the Indo-Pacific, where species richness is very high, the number of species within the same herbivorous group is likely to be important, whereas in areas such as the Caribbean, Brazil or Tropical Eastern Pacific, there could be only a very limited number of species within this group at the regional level [91]. Also, fishing is not the only potential stress factor that needs to be considered when addressing coral reefs resilience to climate change. Other major stressors such as sedimentation, nutrient or other pollutant runoff, are more rarely considered in coral reef management, despite their high impact on coral reef ecosystems worldwide [92], [93]. In a recent study based on a survey of expert’s opinions and scientific literature, fishing pressure was actually ranked last among the 11 factors perceived as the most important to be incorporated into management plans for preserving coral-reef resilience in the context of climate change [87].

### Conclusion

Besides a high social cost in a region where small-scale fishing represents an important matter for both rural and urban populations, an exclusive protection of all herbivorous fish species in New Caledonia would not be capable of mitigating climate change effects on coral reefs. Although herbivorous species are not clearly affected by fishing at the present day, our results suggest that current fishing pressure still has an impact on carnivorous fish, which may ultimately lead to trophic cascade effects in the future [84]. Therefore, in the most heavily fished areas of the country (i.e., outside UNESCO areas), the status of carnivorous fish and key large fished herbivorous, such as the 12 species of large fished macroalgae feeders described in this study, might be monitored as part of a precautionary approach. Conversely, in the sparsely populated UNESCO areas, fishing does not currently appear as the major threat on coral reef communities as compared to other stress factors operating at a large scale in the country, such as the mining industry, which receives minor attention in management schemes as compared to fishing [94]. Mining exploitation and prospecting have resulted in the destruction of land vegetation and soil scraping in large areas of the main island since 1877 (now covering 1,500 km^2^), leading to uncontrolled terrigenous inputs to coastal waters [95] and threatening coastal coral ecosystems [11], [12]. In this respect, the registration of New Caledonia reefs and lagoons to the UNESCO Heritage list provides an appropriate opportunity for promoting integrated coastal zone management schemes that would address all potential stress factors through concerted spatially-explicit regulations of coastal activities.

## Materials and Methods

### New Caledonia Case Study (phase 1)

The list of herbivorous fish species from New Caledonia was extracted from the most recent update of fish diversity in the area [22] and the species diet descriptions based on extensive stomach content analyses conducted by the IRD [96] and/or information from Fishbase [97]. Only strictly herbivorous species (i.e., species for which algae are an essential component of diet, as indicated by their consistent dominance in fish stomach contents), and species known to be well detected by underwater visual census (UVC), were retained (Appendix S1a). Herbivorous fish may be grouped within a number of feeding strategies [56], [85]. Since species composition is usually less stable through time or space than functional composition [98], our approach focused on groups of fish defined based on 1) the type of algae consumed, 2) species size, and 3) species fishing status (Table 1). The type of algae consumed were either microalgae (including microscopic and filamentous algae, i.e., “turf”), macroalgae (including all erect macroscopic algae except filamentous algae), or both. Species size was either small (maximum standard length SL ≤30 cm), or large (maximum SL ≥31 cm). Species were assigned as fished or unfished based on detailed descriptions of local fisheries [61], [99], [100].

Estimates of hard living coral cover (%), macroalgal cover (%), and the biomass, density, diversity and size of herbivorous fish from the groups defined above, were obtained from 14 sites spanning the entire archipelago of New Caledonia. Data was compiled from different UVC surveys conducted between 2004 and 2008. All fish were recorded and sized (to the nearest cm) by distance sampling along 50 m long transects. Length-weight relationships from [101] were used to estimate fish biomass. More details on sampling methods used are provided in [102] for the Beautemps-Beaupré, Bourail, Merlet, Northern lagoon, Ouvéa Island, Pines Island, and Southern lagoon sites, and in [24] and [66] for the Aboré, Koné, Lifou Island, Moindou, Ouassé, and Thio sites (Fig. 1; Appendix S1b). In each of the 14 sites, one to four biotopes were sampled: fringing reef, lagoon patch reef, inner barrier reef, and/or outer barrier reef, resulting in a data set comprising 288 stations from 42 surveyed areas (Table 2). Allocation of sampling effort (i.e., number of stations per site) was based on habitat variability and logistics, and is detailed in [102].

The 14 sites were characterized by different levels of protection status and anthropogenic pressure. A fishing ban had been effectively enforced in Merlet reef, Aboré reef, and Bourail site in 1970, 1988 and 1996, respectively. The remaining 11 sites were not submitted to any kind of protection at the time of sampling. Six of them were registered at the UNESCO World Heritage list in July 2008, given their high biological diversity and near-pristine conditions (i.e., low human disturbances). These six areas have since then been managed as IUCN category IV Marine Protected Areas. We used a human pressure index as a proxy for fishing pressure in the 14 sites (Table 2). First, the coastal human population was calculated, based on the number of inhabitants residing within the maximum distance traveled by fishing boats from the study sites (20 km or 30 km in the case of Aboré reef [60], [61], [99]). This maximum distance was used to estimate the total fishing area (in km^2^) accessible to the coastal population. Human pressure was estimated by the ratio of human population to fishing area (number of inhabitants.km^−2^) and used to define three fishing pressure categories: “none”, “low” and “moderate”. MPAs that had been implemented at the time of sampling and remote sites with no human pressure were categorized as “none”, sites with human pressure ranging from 2 to 7 inhabitants.km^−2^ were categorized as “low”, and sites with human population ranging from 14 to 57 inhabitants.km^−2^ categorized as “moderate”.

Fish biomass, density and diversity were estimated either as quantitative variables (g.m^−2^, number.m^−2^, and number of species per station, respectively) or as relative variables (% of total fish biomass, density and diversity, and % of herbivorous fish biomass, density and diversity, respectively). Fish size was estimated using the average size of the last quartile for each fish group based on the rapid fishing effects on the size structure of fish populations (e.g., [103], [104]). Two-way analyses of variance (ANOVA) were used to examine the influence of biotope, fishing pressure and their interaction on coral and macroalgal covers, and on the quantitative and relative biomass, density, and diversity of each fish group. When a significant effect of fishing pressure was detected (*P*<0.05), Fisher’s Least Significant Difference (LSD) post-hoc tests were performed to examine differences between “none”, “low” and “moderate” fishing pressures, allowing to identify what level of pressure was sufficient for detecting an effect of fishing on the variables examined. The effect of sites, protection status, and UNESCO labeling were not included in the analyses because these three factors were not independent. Furthermore, the registration at the UNESCO World Heritage list did not reflect the protection status applied to the studied sites at the time of sampling.

Finally, relationships between coral cover and macroalgal cover, and between macroalgal cover and the biomass, density and diversity (quantitative and relative) and size of every group of fish consuming macroalgae, were assessed across all surveyed areas, within each biotope and within each level of fishing pressure, using Pearson correlations. For all analyses, coral cover, macroalgal cover, fish diversity and fish size data were square-root transformed. Fish biomass and fish density data were log(x+1) transformed. All statistical tests were performed using Statistica v.10 (StatSoft Inc., 2011).

### New Caledonia Case Study within a Global Framework (Phase 2)

Results obtained from local data in New Caledonia were challenged in the context of a global literature review. Publications reporting changes in reef benthic communities observed specifically after hurricanes and bleaching events (i.e., the major climate-induced disturbances) in reefs with various herbivorous fish protection status were analyzed, including published observations from New Caledonia. This allowed enlarging the scope of our study in the context of current debate about the ability of local management decisions to mitigate the effects of global-scale sources of coral mortality and phase-shifts in reefs systems [27]–[30].

The recovery of corals after hurricanes and/or bleaching events has been shown to depend locally on the state of reefs before the disturbance (i.e., the initial coral cover), the immediate impact on coral cover (i.e., immediate coral mortality), and time (i.e., the post-disturbance period considered when assessing recovery trajectories) [105], [106]. All the aforementioned parameters were thus taken into account when compiling available published data. Consequently, only publications providing quantitative data on coral cover 1) before (*t*_0_), 2) soon after (*t*_1_), and 3) long after (*t*_2_) the impact of a hurricane and/or a bleaching event were retained. This resulted in a total of 27 publications and 36 case studies, among which two were from New Caledonia (Table 4; Appendix S2).

When available, data on macroalgal cover at *t*_0_ and *t*_2_ were also extracted from the selected publications. This allowed evaluating the development of macroalgae relative to corals after climate-induced disturbances, used as a proxy of the potential occurrence of coral-macroalgae phase shifts, i.e., the alternate state likely regulated by herbivorous fish grazing. For the specific need of this survey, macroalgae were considered as growing over corals after a disturbance on a specific reef if 1) macroalgal cover at *t*_2_ was superior to hard living coral cover at *t*_2_, and 2) macroalgal cover increased after the disturbance (*t*_2_) relative to observations made before the disturbance (*t*_0_). The term macroalgae here refers to all erect macroscopic algae except filamentous algae. Although filamentous algae often provide a preliminary substrate for the development of macroalgae on degraded reefs [107], the focus of this analysis has been restricted to macroscopic erect algae since their dominance is generally considered as the most common alternate state of reef degradation in the absence of herbivore regulation [17], [108]. Furthermore, quantitative estimations of filamentous algae cover are rare in the literature, and were not available in most case studies included.

A quantitative index of coral recovery and a qualitative index of macroalgal development relative to corals observed after hurricanes and/or bleaching events were determined based on the assumptions described above. The coral recovery index (*CR*) was calculated as follows:
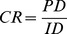
with *PD*: post-disturbance estimate of coral growth, and *ID*: estimate of the initial decline in coral cover observed immediately after the hurricane and/or bleaching event. *PD* and *ID* indexes were calculated as follows:



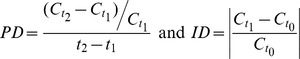
with *C*: coral cover (%) as provided in the selected publications (see Appendix S2).

The macroalgal relative development index (*MD*) was determined as follows:
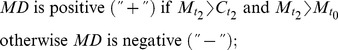
with *M*: macroalgal cover (%) as provided in the selected publications (see Appendix S2).

Values of *CR* and *MD* were then mapped as a function of the protection status of herbivorous fish, defined using two categories: MPAs (i.e., areas where fishing was either banned or strongly restricted) vs. open fishing areas. This allowed identifying potential geographical patterns on coral recovery and macroalgal development relative to corals after hurricanes and/or bleaching events at a global scale. The effect of herbivorous fish protection on *CR* quantitative values and *MD* levels was then statistically tested using non-parametric Mann-Whitney procedure. In addition, the number of case studies for which corals increased or decreased in abundance (*CR*>0 or *CR*<0, respectively) and for which macroalgae developed or not relative to corals at *t*_2_ (*MD* = “+” or “−”, respectively) were plotted as a function of protection status. Finally, the relationships between *CR* and 1) the pre-disturbance coral cover (*C_t0_*) 2) the pre-disturbance macroalgal cover (*M_t0_*), 3) the initial decline in coral cover observed immediately after the disturbance (*ID*), 4) the post-disturbance estimate of coral growth (*PD*), and 5) the time during which coral recovery was measured (*t_2_*-*t_1_*), were tested using linear regressions.

## Supporting Information

Appendix S1 **Data used for New Caledonia case study analysis.** a) List of herbivorous fish species with information on their diet and fishing status. b) Average values of benthic cover and fish variables in each site. c) Average values and results of statistical comparisons (*F* statistic, *P* value) between biotopes and levels of fishing pressure.
(XLSX)

Appendix S2 **Literature data used for global scale analysis.** Values of coral cover and macroalgal cover were extracted from 27 publications including 36 case studies. “*t_0_*” refers to a period before the climatic disturbance, “*t_1_*” soon after, and “*t_2_*” several months and years after. For each case study, the exact year or month at which observations were obtained is indicated in subscript. Final values of *CR* and *MD* indices are provided in Table 4. Calculation formulae are described in the Materials and Methods section. “MPA” = Marine Protected Area (fishing banned or restricted), “open” = open area (unprotected, fished), and “na” = not available.
(PDF)

## References

[1] Walther GR, Post E, Convey P, Menzel A, Parmesan C, et al (2002) Ecological responses to recent climate change. Nature 416: 389–395. 11919621 10.1038/416389a

[2] Hoegh-Guldberg O, Mumby PJ, Hooten AJ, Steneck RS, Greenfield P, et al (2007) Coral reefs under rapid climate change and ocean acidification. Science 318: 1737–1742. 18079392 10.1126/science.1152509

[3] Bellwood DR, Hughes TP, Folke C, Nyström M (2004) Confronting the coral reef crisis. Nature 429: 827–833. 15215854 10.1038/nature02691

[4] Keller B, Gleason D, McLeod E, Woodley C, Airamé S, et al (2009) Climate Change, Coral Reef Ecosystems, and Management Options for Marine Protected Areas. Environmental Management 44: 1069–1088. 19636605 10.1007/s00267-009-9346-0 PMC2791481

[5] Hughes TP, Baird AH, Bellwood DR, Card M, Connolly SR, et al (2003) Climate change, human impacts, and the resilience of coral reefs. Science 301: 929–933. 12920289 10.1126/science.1085046

[6] McManus JW, Menez LAB, Kesner-Reyes KN, Vergara SG, Ablan MC (2000) Coral-reef fishing and coral-algal phase shifts: implications for global reef status. ICES Journal of Marine Science 57: 572–578.

[7] Knowlton N, Lang JC, Keller BD (1990) Case study of a natural population collapse: post-hurricane predation on Jamaica staghorne corals. Smithsonian Contributions to the Marine Sciences 31: 1–25.

[8] Cameron AM, Endean R, DeVantier LM (1991) Predation on massive corals: are devastating population outbreaks of *Acanthaster planci* novel events? Marine Ecology Progress Series 75: 251–268.

[9] Aronson RB, Precht WF (2001) White-band disease and the changing face of Caribbean coral reefs. Hydrobiologia 460: 25–38.

[10] Raymundo LJ, Halford AR, Maypa AP, Kerr AM (2009) Functionally diverse reef-fish communities ameliorate coral disease. Proceedings of the National Academy of Science USA 106: 17067–17070. 10.1073/pnas.0900365106 PMC2761369 19805081

[11] McClanahan TR, Obura D (1997) Sedimentation effects on shallow coral communities in Kenya. Journal of Experimental Marine Biology and Ecology 209: 103–122.

[12] Bruno JF, Petes LE, Harvell CD, Hettinger A (2003) Nutrient enrichment can increase the severity of coral diseases. Ecology Letters 6: 41056–41061.

[13] Wilkinson C (2008) Status of coral reefs of the world. Townsville: Global Coral Reef Monitoring Network Reef and Rainforest research Center. 304 p.

[14] Hoegh-Guldberg O (1999) Climate change, coral bleaching and the future of the world’s coral reefs. Marine and Freshwater Research 50: 839–866.

[15] Sheppard CRC (2003) Predicted recurrences of mass coral mortality in the Indian Ocean. Nature 425: 294–297. 13679917 10.1038/nature01987

[16] Burke L, Reytar K, Spalding M, Perry A (2011) Reef at risk revisited. Washington DC: World Resources Institute. 115 p.

[17] McManus JW, Polsenberg JF (2004) Coral-algal phase shifts on coral reefs: ecological and environmental aspects. Progress in Oceanography 60: 263–279.

[18] Noström AV, Nystrom M, Lokrantz J, Folke C (2009) Alternative states on coral reefs : beyond coral-macroalgal phase shifts. Marine Ecology Progress Series 376: 295–306.

[19] Andréfouët S, Cabioch G, Flamand B, Pelletier B (2009) A reappraisal of the diversity of geomorphological and genetic processes of New Caledonian coral reefs: a synthesis from optical remote sensing, coring and acoustic multibeam observations. Coral Reefs 28: 691–707.

[20] Pichon M (2006) Scleractinia of New Caledonia: checklist of reef dwelling species. In: Nouméa : Documents Scientifiques et Techniques de l’IRD Payri CE, Richer de Forges B, editors. Compendium of marine species from New Caledonia. II7: 147–155.

[21] Payri CE (2006) Revised checklist of marine algae (Chlorophyta, Rhodophyta and Ochrophyta) and seagrasses (Marine Angiosperma) of New Caledonia. In: Nouméa: Documents Scientifiques et Techniques de l’IRD Payri CE, Richer de Forges B, editors. Compendium of marine species from New Caledonia. II7: 93–110.

[22] Fricke R, Kulbicki M, Wantiez L (2011) Checklist of the shore fishes of New Caledonia, and their distribution in the Southwestwantiex Pacific Ocean (Pisces). Stuttgarter Beiträge zur Naturkunde A Neue Serie 4: 341–363.

[23] Wantiez L, Chateau O, Le Mouellic S (2006) Initial and mid-term impacts of cyclone Erica on coral reef fish communities and habitat in the South Lagoon Marine Park of New Caledonia. Journal of the Marine Biological Association of the United-Kingdom 86: 1229–1236.

[24] Guillemot N, Chabanet P, Le Pape O (2010) Cyclone effects on coral reef habitats in New Caledonia (South Pacific). Coral Reefs 29: 445–453.

[25] Richer de Forges B (1998) La diversité du benthos de Nouvelle-Calédonie: de l’espèce à la notion de patrimoine. PhD dissertation. Paris: Muséum National d’Histoire Naturelle.

[26] Hughes TP, Bellwood DR, Floke CS, McCook LJ, Pandolfi JM (2007) No-take areas, herbivory and coral reef resilience. Trends in Ecology and Evolution 22: 1–3. 17070963 10.1016/j.tree.2006.10.009

[27] Aronson RB, Precht WF (2006) Conservation, precaution, and Caribbean reefs. Coral Reefs 25: 441–450.

[28] Graham NAJ, McClanahan TR, MacNeil MA, Wilson SK, Polunin NVC, et al (2008) Climate warming, Marine Protected Areas and the ocean-scale integrity of coral reef ecosystems. PLoS ONE 3: e3039. 18728776 10.1371/journal.pone.0003039 PMC2516599

[29] Steneck R, Paris C, Arnold S, Ablan-Lagman M, Alcala A, et al (2009) Thinking and managing outside the box: coalescing connectivity networks to build region-wide resilience in coral reef ecosystems. Coral Reefs 28: 367–378.

[30] Côté IM, Darling ES (2010) Rethinking ecosystem resilience in the face of climate change. PLoS Biology 8: e1000438. 20668536 10.1371/journal.pbio.1000438 PMC2910654

[31] Wilkinson C, Souter D (2008) Status of Caribbean coral reefs after bleaching and hurricanes in 2005. Townsville: Global Coral Reef Monitoring Network Reef and Rainforest Research Center. 152 p.

[32] Aronson RB, Precht WF, Macintyre IG, Murdoch TJT (2000) Coral bleach out in Belize. Nature 405: 36. 10811207 10.1038/35011132

[33] Somerfield PJ, Jaap WC, Clarke KR, Callahan M, Hackett K, et al (2008) Changes in coral reef communities among the Florida Keys, 1996–2003. Coral Reefs 27: 951–965.

[34] Hughes TP (1994) Catastrophes, phase shifts, and large-scale degradation of a Caribbean coral reef. Science 265: 1547–1551. 17801530 10.1126/science.265.5178.1547

[35] Idjadi JA, Lee SC, Bruno JF, Precht WF, Allen-Requa L, et al (2006) Rapid phase shift reversal on a Jamaican coral reef. Coral Reefs 25: 209–211.

[36] Shulman MJ, Robertson DR (1996) Changes in the coral reefs of San Blas, Caribbean Panama: 1983 to 1990. Coral Reefs 15: 231–236.

[37] Hawkins JP, Roberts CM, Dytham C, Schelten C (2006) Effects of habitat characteristics and sedimentation on performance of marine reserves in St. Lucia. Biological Conservation 127: 487–499.

[38] Bythell JC, Hillis-Starr ZM, Rogers CS (2000) Local variability but landscape stability in coral reef communities following repeated hurricane impacts. Marine Ecology Progress Series 204: 93–100.

[39] Rogers CS, McLain LN, Tobias CR (1991) Effects of hurricane Hugo (1989) on a coral reef in St John, US Virgin Islands. Marine Ecology Progress Series 78: 189–199.

[40] Riegl B (2002) Effects of the 1996 and 1998 positive sea-surface temperature anomalies on corals, coral diseases and fish in the Arabian Gulf (Dubai, UAE). Marine Biology 140: 29–40.

[41] Burt J, Batholomew A, Usseglio P (2008) Recovery of corals a decade after a bleaching event in Dubai, United Arab Emirates. Marine Biology 154: 27–36.

[42] McClanahan TR, Maina J, Pet-Soele L (2002) Effects of the 1998 coral mortality event on Kenyan coral reefs and fisheries. Ambio 31: 543–550. 12572820

[43] McClanahan TR (2000) Bleaching damage and recovery potential of Maldivian coral reefs. Marine Pollution Bulletin 40: 587–597.

[44] Lasagna R, Albertelli G, Giovannetti E, Grondona M, Milani A, et al (2008) Status of Maldivian reefs eight years after the 1998 coral mass mortality. Chemistry and Ecology 24: 67–72.

[45] Ledlie MH, Graham NAJ, Bythell JC, Wilson SK, Jennings S, et al (2007) Phase shifts and the role of herbivory in the resilience of coral reefs. Coral Reefs 26: 641–653.

[46] Diaz-Pulido G, McCook MJ, Dove S, Berkelmans R, Roff G, et al (2009) Doom and boom on a resilient reef: climate change, algal overgrowth and coral recovery. PLoS ONE 4: e5239. 19384423 10.1371/journal.pone.0005239 PMC2668766

[47] Halford A, Cheal AJ, Ryan D, Williams DM (2004) Resilience to large-scale disturbance in coral and fish assemblages on the Great Barrier Reef. Ecology 85: 1892–1905.

[48] Hoegh-Guldberg O, Fine M, Skirving W, Johnstone R, Dove S, et al (2005) Coral bleaching following wintry weather. Limnology and Oceanography 50: 265–271.

[49] Connell JH, Hughes TP, Wallace CC (1997) A 30-year study of coral abundance, recruitment and disturbance at several scales in space and time. Ecological Monographs 67: 461–488.

[50] Smith LD, Gilmour JP, Heyward AJ (2008) Resilience of coral communities on an isolated system of reefs following catastrophic mass-bleaching. Coral Reefs 27: 197–205.

[51] Coles SL, Brown EK (2007) Twenty-five years of change in coral coverage on a hurricane impacted reef in Hawai’i: the importance of recruitment. Coral Reefs 26: 705–717.

[52] Dollar SJ, Tribble GW (1993) Recurrent storm disturbance and recovery: a long-term study of coral communities in Hawaii. Coral Reefs 12: 223–233.

[53] Brown BE (1990) Suharsono (1990) Damage and recovery of coral reefs affected by El Niño related seawater warming in the Thousand Islands, Indonesia. Coral Reefs 8: 163–170.

[54] Golbuu Y, Victor S, Penland L, Idip D Jr, Emaurois C, et al (2007) Palau’s coral reefs show differential habitat recovery following the 1998-bleaching event. Coral Reefs 26: 319–332.

[55] Adjeroud M, Michonneau F, Edmunds PJ, Chancerelle Y, Lison de Loma R, et al (2009) Recurrent disturbances, recovery trajectories, and resilience of coral assemblages on a South Central pacific Reef. Coral Reefs 28: 775–780.

[56] Clements KD, Raubenheimer D, Choat JH (2009) Nutritional ecology of marine herbivorous fishes: ten years on. Functional Ecology 23: 79–92.

[57] Hoey AS, Bellwood DR (2010) Among-habitat variation in herbivory on *Sargassum* spp. on a mid-shelf reef in the northern Great barrier Reef. Marine Biology 157: 189–200.

[58] Vergés A, Bennett S, Bellwood DR (2012) Diversity among macroalgae-consuming fishes on coral reefs: a transcontinental comparison. PLoS ONE 7: e45543. 23029083 10.1371/journal.pone.0045543 PMC3447800

[59] Mellin C, Kulbicki M. Ponton D (2007) Seasonal patterns in habitat use of reef fish juveniles in the south-west lagoon of New Caledonia. Estuarine Coastal and Shelf Science 75: 481–491.

[60] Jollit I, Léopold M, Guillemot N, David G, Chabanet P, et al (2010) Geographical aspects of informal reef fishery systems in New Caledonia. Marine Pollution Bulletin 61: 585–597. 20667555 10.1016/j.marpolbul.2010.06.033

[61] Guillemot N, Léopold M, Chabanet P, Cuif M (2009) Characterization and management of informal fisheries confronted with socio-economic changes in New Caledonia (South Pacific). Fisheries Research 98: 51–61.

[62] David G, Léopold M, Dumas PS, Ferraris J, Herrenschmidt JB, et al (2010) Integrated coastal zone management perspectives to ensure the sustainability of coral reefs in New Caledonia. Marine Pollution Bulletin 61: 323–334. 20655550 10.1016/j.marpolbul.2010.06.020

[63] Kulbicki M (1997) Bilan de 10 ans de recherche (1985–1995) par l’ORSTOM sur la structure des communautés des poissons lagonaires et récifaux en Nouvelle-Calédonie. Cybium 21: 47–79.

[64] Letourneur Y, Kulbicki M, Labrosse P (2000) Commercial demersal fish stock assessment of the Northern New Caledonian lagoon. 1. Coral reef fish communities. Aquatic Living Resources 13: 65–76.

[65] Kulbicki M, Sarramégna S, Letourneur Y, Wantiez L, Galzin R, et al (2007) Short-term temporal changes in the structure of a coral reef fish assemblage in a New Caledonian protected area: relative influence of opening to fishing. Journal of Experimental Marine Biology and Ecology 353: 145–163.

[66] Chabanet P, Guillemot N, Kulbicki M, Vigliola L, Sarramegna S (2010) Baseline study of the spatio-temporal patterns of reef fish assemblages prior to a major mining project in New Caledonia (South Pacific). Marine Pollution Bulletin 61: 598–611. 20637479 10.1016/j.marpolbul.2010.06.032

[67] Sale PF, editor (2002) Coral reef fishes: dynamics and diversity in a complex ecosystem. San Diego: Academic Press. 549 p.

[68] Choat H, Robertson R (2002) Age-based studies on coral reef fishes. In: Sale PF (editor) Coral reef fishes: dynamics and diversity in a complex ecosystem. San Diego: Academic Press. 57–80.

[69] Rossier O, Kulbicki M (2000) A comparison of fish assemblages from two types of algae beds and coral reefs in the South-West lagoon of New Caledonia. Cybium 24 3–26.

[70] Williams ID, Polunin NVC (2001) Large-scale associations between macroalgae cover and grazer biomass on mid-depth reefs in the Caribbean. Coral Reefs 19: 358–366.

[71] Williams ID, Polunin NVC, Hendrick VJ (2001) Limits to grazing by herbivorous fishes and the impact of low coral cover on macroalgal abundance on a coral reef in Belize. Marine Ecology Progress Series 222: 187–196.

[72] Mumby PJ (2006) The impact of exploited grazers (Scaridae) on the dynamics of Caribbean coral reefs. Ecological Applications 16: 747–769. 16711060 10.1890/1051-0761(2006)016[0747:tioegs]2.0.co;2

[73] Newman MJH, Paredes GA, Sala E, Jackson JBC (2006) Structure of Caribbean coral reef communities across a large gradient of fish biomass. Ecology Letters 9: 1216–1227. 17040324 10.1111/j.1461-0248.2006.00976.x

[74] Hughes TP, Rodriguez MJ, Bellwood DR, Ceccarelli D, Hoegh-Guldberg O, et al (2007) Phase shifts, herbivory and the resilience of coral reefs to climate change. Current Biology 17: 1–6. 17291763 10.1016/j.cub.2006.12.049

[75] Tenório MMB, Le Borgne R, Rodier M, Neveux J (2005) The impact of terrigenous inputs in the bay of Ouinné (New Caledonia) phytoplankton communities: a spectrofluorometric and microscopic approach. Estuarine Coastal and Shelf Science 64: 531–545.

[76] Jacquet S, Delesalle B, Torreton JP, Blanchot J (2006) Response of phytoplankton communities to increased anthropogenic influences (southwestern lagoon, New Caledonia). Marine Ecology Progress Series 320: 65–78.

[77] Darling ES, McClanahan TR, Côté IM (2010) Combined effects of two stressors on Kenyan coral reefs are additive or antagonistic, not synergistic. Conservation Letters 3: 122–133.

[78] McClanahan TR (2008) Response of the coral reef benthos and herbivory to fishery closure management and the 1998 ENSO disturbance. Oecologia 155: 169–177. 17972104 10.1007/s00442-007-0890-0

[79] Jones GP, McCormick MI, Srinivasan M, Eagle JV (2004) Coral decline threatens fish biodiversity in marine reserves. Proceedings of the National Academy of Science USA 101: 8251–8253. 10.1073/pnas.0401277101 PMC419589 15150414

[80] Kramer KL, Heck KL (2007) Top-down trophic shifts in Florida Keys patch reef marine protected areas. Marine Ecology Progress Series 349: 111–123.

[81] Mora C (2008) A clear human footprint in the coral reefs of the Caribbean. Proceedings of the Royal Society of London B Biological Sciences 275: 767–773. 10.1098/rspb.2007.1472 PMC2596901 18182370

[82] Mumby PJ, Harborne AR (2010) Marine reserves enhance the recovery of corals on Caribbean Reefs. PLoS ONE 5: e8657. 20066158 10.1371/journal.pone.0008657 PMC2799675

[83] Selig ER, Bruno JF (2010) A global analysis of the effectiveness of Marine Protected Areas in preventing coral loss. PLoS ONE 5: e9278. 20174644 10.1371/journal.pone.0009278 PMC2822846

[84] Mumby PJ, Dahlgren CP, Harborne AR, Kappel CV, Micheli F, et al (2006) Fishing, trophic cascades, and the process of grazing on coral reefs. Science 311: 98–101. 16400152 10.1126/science.1121129

[85] Burkepile DE, Hay ME (2010) Impact of herbivore identity on algal succession and coral growth on a Caribbean reef. PLoS ONE 5: e8963. 20126450 10.1371/journal.pone.0008963 PMC2813280

[86] Rasher DB, Engel S, Bonito V, Fraser GJ, Montoya JP, et al (2012) Effects of herbivory, nutrients, and reef protection on algal proliferation and coral growth on a tropical reef. Oecologia 169: 187–198. 22038059 10.1007/s00442-011-2174-y PMC3377479

[87] McClanahan TR, Donner SD, Maynard JA, MacNeil MA, Graham NAJ, et al (2012) Prioritizing key resilience factors to support coral reef management in a changing climate. PLoS ONE 7: e42884. 22952618 10.1371/journal.pone.0042884 PMC3430673

[88] Jameson SC, Tupper MH, Ridley JM (2002) The three screen doors: can marine “protected” areas be effective? Marine Pollution Bulletin 44: 1177–1183. 12523516 10.1016/s0025-326x(02)00258-8

[89] Cheal AJ, MacNeil MA, Cripps E, Emslie MJ, Jonker M, et al (2010) Coral-macroalgal phase shifts or reef resilience: links with diversity and functional roles of herbivorous fishes on the Great Barrier Reef. Coral Reefs 29: 1005–1015.

[90] Roff G, Mumby PJ (2012) Global disparity in the resilience of coral reefs. Trends in Ecology and Evolution 27 404–413. 22658876 10.1016/j.tree.2012.04.007

[91] Floeter S, Behrens M, Ferreira C, Paddack M, Horn M (2005) Geographical gradients of marine herbivorous fishes: patterns and processes. Marine Biology 147: 1435–1447.

[92] Fung T, Seymour RM, Johnson CR (2011) Alternative stable states and phase shifts in coral reefs under anthropogenic stress. Ecology 92: 967–982. 21661558 10.1890/10-0378.1

[93] Fenner D (2012) Challenges for managing fisheries on diverse coral reefs. Diversity 4: 105–106.

[94] Pascal M, Richer de Forges B, Le Guyader H, Simberloff D (2008) Mining and other threats to the New Caledonia biodiversity hotspot. Conservation Biology 22: 498–499. 18402591 10.1111/j.1523-1739.2008.00889.x

[95] Bird ECF, Dubois JP, Iltis JA (1984) The impacts of opencast mining on the rivers and coasts of New Caledonia. Tokyo: The United Nations University. 64 p.

[96] Kulbicki M (2006) Écologie des poisons lagonaires de Nouvelle-Calédonie. PhD dissertation. Perpignan: École Pratique des Hautes Études.

[97] Froese R, Pauly D, editors (2012) FishBase. World Wide Web electronic publication. Available: http://www.fishbase.org/. Accessed 2012 May 15.

[98] Loreau M, Naeem S, Inchausti P, Bengtsson J, Grime JP, et al (2001) Biodiversity and ecosystem functioning: current knowledge and future challenges. Science 249: 804–808. 10.1126/science.1064088 11679658

[99] Léopold M, Ferraris J, Labrosse P (2004) Assessment of the reliability of fish consumption as an indicator of reef fish catches in small Pacific islands: the example of Ouvéa Island in New Caledonia. Aquatic Living Resources 17: 119–127.

[100] Labrosse P, Letourneur Y, Kulbicki M, Paddon JR (2000) Fish stock assessment of the northern New Caledonian lagoons. 3. Fishing pressure, potential yields and impact on management options. Aquatic Living Resources 13: 91–98.

[101] Kulbicki M, Guillemot N, Armand M (2005) A general approach to length-weight relationships for Pacific lagoon fishes. Cybium 29(3): 235–252.

[102] Andréfouët S, Wantiez L (2010) Characterizing the diversity of coral reef habitats and fish communities found in UNESCO World Heritage site: the strategy developed for Lagoons of New Caledonia. Marine Pollution Bulletin 61: 612–621. 20674947 10.1016/j.marpolbul.2010.06.031

[103] Jennings S, Kaiser MJ (1998) The effects of fishing on marine ecosystems. Advances in Marine Biology 34: 201–352.

[104] Dulvy NK, Polunin NVC, Mill AC, Graham NAJ (2004) Size structure change in lightly exploited coral reef fish communities: evidence for weak indirect effects. Canadian Journal of Fisheries and Aquatic Sciences 61: 466–475.

[105] Baker AC, Glynn PW, Riegl B (2008) Climate change and coral reef bleaching: an ecological assessment of long-term impacts, recovery trends and future outlook. Estuarine Coastal and Shelf Science 80: 435–471.

[106] Anthony KRN, Hoogenboom MO, Maynard JA, Grottoli AG, Middlebrook R (2009) Energetics approach to predicting mortality risk from environmental stress: a case study of coral bleaching. Functional Ecology 23: 539–550.

[107] McClanahan TR (1997) Primary succession of coral-reef algae: differing patterns in fished versus unfished reefs. Journal of Experimental Marine Biology and Ecology 218: 77–102.

[108] Bruno JF, Sweatman H, Precht WF, Selig ER, Schutte VGW (2009) Assessing evidence of phase shifts from coral to macroalgal dominance on coral reefs. Ecology 90: 1478–1484. 19569362 10.1890/08-1781.1

