# Supplementary material for: Does Herbivorous Fish Protection Really Improve Coral Reef Resilience? A Case Study from New Caledonia (South Pacific)
Source: PLoS One. 2013 Apr 5;8(4):e60564. doi: 10.1371/journal.pone.0060564 (PMC3618332; doi:10.1371/journal.pone.0060564)
Supplement: Appendix S2 — Literature data used for global scale analysis. Values of coral cover and macroalgal cover were extracted from 27 publications including 36 case studies. “t0” refers to a period before the climatic disturbance, “t1” soon after, and “t2” several months and years after. For each case study, the exact year or month at which observations were obtained is indicated in subscript. Final values of CR and MD indices are provided in Table 4. Calculation formulae are described in the Materials and Methods section. “MPA” = Marine Protected Area (fishing banned or restricted), “open” = open area (unprotected, fished), and “na” = not available. (PDF) [file pone.0060564.s002.pdf]

Place	Hurricane (year)	Bleaching (year)	Protection status	Coral cover (%) initial (t_0)	Coral cover (%) after (t_1,t_2)	Macroalgae cover (%) initial (t_0)	Macroalgae cover (%) after (t_2)	Reference
ATLANTIC OCEAN								
Belize, global assessment	1998	1998	MPA	30_{1997}	12_{1999}<br>9_{2006}	18_{1997}	30_{2006}	[31]
Belize, global assessment	1998	1998	open	28_{1997}	11_{1999}<br>12_{2006}	12_{1997}	26_{2006}	[31]
Belize, Channel Cay	1998	1998	open	41_{1998}	12_{1999}<br>5_{2000}	na	na	[32]
Florida Keys (26 sites), patch and shallow offshore reefs	1998<br>1999	1997<br>1998	open	17_{1996}	13_{2000}<br>15_{2003}	na	na	[33]
Jamaica,								
Discovery Bay	1980	1987, 1989<br>1990	open	60_{1977}	15_{1990}<br>10_{1995}	3_{1977}	80_{1995}	[34]
Dairy Bull reef	1980	1987, 1989<br>1990	open	55_{1970}	23_{1995}<br>54_{2004}	5_{1970}	6_{2004}	[35]
Panama, San Blas Islands		1983	open	44_{1983}	25_{1987}<br>20_{1990}	2_{1983}	30_{1990}	[36]
St Lucia	1999		MPA	39_{1998}	18_{2000}<br>20_{2002}	12_{1998}	18_{2002}	[37]
St Lucia	1999		open	20_{1998}	14_{2000}<br>15_{2002}	31_{1998}	32_{2002}	[37]
US Virgin Islands								
St Croix, Buck Island (2 sites)	1989,<br>1996(2)		MPA	29_{1989}	27_{1991}<br>31_{1996}	5_{1989}	4_{1996}	[38]
St John, Yawsi Point	September 1989		MPA	20_{Jun89}	13_{Nov89}<br>14_{Nov90}	4_{Jun89}	18_{Nov90}	[39]

Place	Hurricane (year)	Bleaching (year)	Protection status	Coral cover (%) initial (t_0)	Coral cover (%) after (t_1,t_2)	Macroalgae cover (%) initial (t_0)	Macroalgae cover (%) after (t_2)	Reference
RED SEA								
Dubai, Arabian Gulf		1996<br>1998	open	90_{1995}	22_{1998}<br>42_{2006}	na	na	[40,41]
INDIAN OCEAN								
Kenya (4 sites)		1998	open	21_{1997}	11_{1999}<br>14_{2001}	4_{1997}	17_{2001}	[42]
Kenya (3 sites)		1998	MPA	42_{1997}	11_{1999}<br>20_{2001}	4_{1997}	10_{2001}	[42]
Maldives (~30 sites)		1990<br>1998	open	56_{1964}	28_{1992}<br>8_{1999}	na	9_{1999}	[43]
Maldives central atolls (Ari, south Malé, Felidhu)		1998	open	70_{1998}	5_{1999}<br>25_{2006}	5_{1998}	7_{2006}	[44]
Seychelles, Cousin Island		1998	MPA	40_{1994}	9_{1998}<br>1_{2005}	1_{1994}	30_{2005}	[45]
PACIFIC OCEAN								
Australia, Great Barrier Reef								
Middle Island		Jan-Feb 2006	MPA	80_{Feb06}	20_{Aug06}<br>60_{Feb08}	20_{Feb06}	30_{Feb08}	[46]
Halfway Island		Jan-Feb 2006	MPA	90_{Feb06}	30_{Aug06}<br>85_{Feb08}	5_{Feb06}	10_{Feb08}	[46]
Barren Island		Jan-Feb 2006	MPA	80_{Feb06}	30_{Aug06}<br>85_{Feb08}	na	1_{Feb08}	[46]
North Keppel Island		Jan-Feb 2006	MPA	45_{Feb06}	8_{Aug06}<br>10_{Feb08}	35_{Feb06}	60_{Feb08}	[46]

Place	Hurricane (year)	Bleaching (year)	Protection status	Coral cover (%) initial (t_0)	Coral cover (%) after (t_1,t_2)	Macroalgae cover (%) initial (t_0)	Macroalgae cover (%) after (t_2)	Reference
PACIFIC OCEAN, continued								
Australia, Great Barrier Reef, continued								
Capricorn Bunker & Swains sectors	several storms between 1987 & 1989		MPA	85_{1987}	8_{1989}<br>80_{1998}	na	na	[47]
Capricorn Bunker sector Heron Island		August 2003	MPA	80_{Aug03}	30_{Sep03}<br>20_{Jan04}	na	na	[48]
Heron Island, inner flat	1967, 1972, 1976, 1980, 1992		MPA	15_{1965}	14_{1970}<br>0_{1992}	na	na	[49]
Heron Island, exposed pools	〃		MPA	50_{1965}	1_{1970}<br>10_{1992}	na	na	[49]
Heron Island, protected crest	〃		MPA	50_{1965}	70_{1970}<br>10_{1992}	na	na	[49]
Heron Island, exposed crest	〃		MPA	75_{1965}	50_{1970}<br>25_{1992}	na	na	[49]
Australia (NW), Scott Reefs		1998	open	47_{1998}	11_{1999}<br>20_{2003}	1_{1998}	1_{2003}	[50]
Hawai'i, O'ahu (3 sites)	1980, 1982, 1992		open	40_{1980}	16_{1997}<br>18_{2003}	"low" (<10)	"low" (<10)	[51]
Hawai'i, west coast	1980, 1982, 1992(…)		open	57_{1974}	14_{1980}<br>13_{1993}	na	na	[52]

Place	Hurricane (year)	Bleaching (year)	Protection status	Coral cover (%) initial (t_0)	Coral cover (%) after (t_1,t_2)	Macroalgae cover (%) initial (t_0)	Macroalgae cover (%) after (t_2)	Reference
PACIFIC OCEAN, continued								
Indonesia, Thousand Islands								
South Pari		1983	open	22_{1981}	4_{1983}<br>15_{1988}	na	na	[53]
South Tikus		1983	open	26_{1981}	2_{1983}<br>12_{1988}	na	na	[53]
Micronesia, Palau		1998	open	60_{1992}	17_{2001}<br>30_{2004}	na	na	[54]
New Caledonia<br>Southwest lagoon	March 2003		MPA	34_{Mar03}	29_{Apr03}<br>25_{Nov04}	14_{Mar03}	20_{Nov04}	[23]
Northwest lagoon	March 2003		open	15_{2002}	8_{2004}<br>17_{2007}	15_{2002}	13_{2007}	[24]
Polynesia, Moorea	1991	1991, 1994, 2002, 2003	open	50_{1991}	25_{1993}<br>50_{2006}	1_{1991}	1_{2006}	[55]
